# SPAK Deficiency Corrects Pseudohypoaldosteronism II Caused by WNK4 Mutation

**DOI:** 10.1371/journal.pone.0072969

**Published:** 2013-09-11

**Authors:** Pei-Yi Chu, Chih-Jen Cheng, Yi-Chang Wu, Yu-Wei Fang, Tom Chau, Shinichi Uchida, Sei Sasaki, Sung-Sen Yang, Shih-Hua Lin

**Affiliations:** 1 Graduate Institute of Medical Sciences, National Defense Medical Center, Taipei, Taiwan; 2 Division of Nephrology, Department of Medicine, Tri-Service General Hospital, Taipei, Taiwan; 3 Taoyuan Armed Forces General Hospital, Taoyuan, Taiwan; 4 Division of Nephrology, Shin Kong Wu Ho-Su Memorial Hospital, Taipei, Taiwan; 5 Department of Medicine, Providence St. Vincent Medical Center, Portland, Oregon, United States of America; 6 Department of Nephrology, Graduate School of Medicine, Tokyo Medical and Dental University, Tokyo, Japan; INSERM, France

## Abstract

Stimulation of the OSR1 (Oxidative stress-responsive kinase-1)/SPAK [STE20 (sterile 20)/SPS1-related proline/alanine-rich kinase]-NCC (Na^+^-Cl^−^ cotransporter) signaling cascade plays an important role in the WNK [With-No-Lysine (K)] kinase 4 D561A knock-in mouse model of pseudohypoaldosteronism type II (PHA II) characterized by salt-sensitive hypertension and hyperkalemia. The aim of this study was to investigate the respective roles of Osr1 and Spak in the pathogenesis of PHA II *in vivo*. *Wnk4* ^D561A/+^ mice were crossed with kidney tubule-specific (KSP) *Osr1* knockout (KSP-*Osr1* ^−/−^) and *Spak* knockout (*Spak* ^−/−^) mice. Blood pressure, plasma and urine biochemistries, and the relevant protein expression in the kidneys were examined. *Wnk4* ^D561A/+^, KSP-*Osr1* ^−/−^, and *Spak* ^−/−^ mice recapitulated the phenotypes of PHA II, Bartter-like syndrome, and Gitelman syndrome, respectively. *Wnk4* ^D561A/+^.KSP-*Osr1* ^−/−^ remained phenotypically PHA II while *Wnk4* ^D561A/+^.*Spak* ^−/−^ mice became normotensive and lacked the PHA II phenotype. Phosphorylated Spak and Ncc were similarly increased in both *Wnk4* ^D561A/+^ and *Wnk4* ^D561A/+^.KSP-*Osr1* ^−/−^ mice while phosphorylated Ncc normalized in *Wnk4* ^D561A/+^.*Spak* ^−/−^ mice. Furthermore, *Wnk4* ^D561A/+^.KSP-*Osr1* ^−/−^ mice exhibited exaggerated salt excretion in response to thiazide diuretics while *Wnk4* ^D561A/+^.*Spak* ^−/−^ mice exhibited normal responses. *Wnk4^D561A/+^.Spak* ^−*/*−^.*KSP-Osr1* ^−*/*−^ triple mutant mice had low blood pressure and diminished phosphorylated Ncc. Both SPAK and OSR1 are important in the maintenance of blood pressure but activation of SPAK-NCC plays the dominant role in PHA II. SPAK may be a therapeutic target for disorders with salt-sensitive hypertension related to WNK4 activation.

## Introduction

The kidneys are vital to salt balance and blood pressure regulation. The thiazide-sensitive sodium chloride cotransporter (Na^+^-Cl^−^ cotransporter, NCC) in the distal convoluted tubule (DCT) is responsible for 5-10% of total filtered Na^+^ reabsorption and regulates Na^+^ homeostasis and blood pressure.[1], [2] Inactivating mutations of the *NCC* gene *SLC12A3* cause autosomal recessive Gitelman syndrome (GS) characterized by renal salt wasting with secondary hyperreninemia and hyperaldosteronism, renal potassium (K^+^) wasting with chronic hypokalemia, metabolic alkalosis, and hypocalciuria.[3]–[10] In contrast, autosomal dominant Gordon syndrome, also called familial hyperkalemic hypertension and pseudohypoaldosteronism type II (PHA II), is the mirror image of GS and featured with thiazide-correctable salt-sensitive hypertension with low plasma renin activity (PRA) and aldosterone levels and hyperkalemic metabolic acidosis.[11]–[14] It is caused by gain of NCC function from well-described mutations in the WNK [With-No-Lysine (K)] 1 and 4 kinase genes[15] and newly-reported mutations in Kelch-like 3 or cullin 3.[16]–[18]

Previous *in vitro* studies have demonstrated SPAK [STE20 (sterile 20)/SPS1-related proline/alanine-rich kinase] and OSR1 (Oxidative stress-responsive kinase-1) as downstream substrates of WNK kinases.[19]–[22] They belong to the same germinal center kinase VI family and share high sequence homology in their N-terminal catalytic and C-terminal regulatory domains. In the kidneys, the distributions of SPAK and OSR1 virtually overlap in the distal nephron.[23]–[25] Activated SPAK/OSR1 subsequently phosphorylate and activate several cation-chloride cotransporters (CCC), including Na^+^-K^+^-2Cl^−^ cotransporter isoform 1 and 2 (NKCC1, NKCC2), NCC, and potassium chloride cotransporter.[26]–[28] The WNK-SPAK/OSR-CCC signaling cascade plays a pivotal role in volume regulation and blood pressure control.[27], [29]–[31] In the mutant *Wnk4* D561A mouse model of PHA II, constitutively active *Wnk4* increased phosphorylation of Spak/Osr1 and lead to Ncc overactivity in the DCT.[32] In contrast, *Wnk4* hypomorphic or deficient mice exhibit decreased phosphorylated (p-)Spak/Osr1 and p-Ncc and hypotension. In the next step of the cascade, knock-out *Spak* and *Osr1* mice exhibit hypotension with decreased abundance of p-Nkcc and p-Ncc.[24], [29], [33]

The individual roles of Spak and Osr1 in the pathogenesis of PHA II *in vivo* have not been easily illucidated as they are coincidentally expressed in the DCT and thick ascending limb (TAL). [29] To study them individually, we abolished the expression of Spak or Osr1 specifically in the distal nephron in a mouse model of PHA II by crossing constitutively active *Wnk4* ^D561A/+^ knockin (KI) mice with kidney tubule-specific (KSP)-*Osr1* knockout (KSP*-Osr1* ^−*/*−^) or *Spak* knockout (*Spak* ^−/−^) mice.[29], [33] The results to be reported indicated that *Wnk4* ^D561A/+^.KSP*-Osr1* ^−*/*−^ mice still exhibited the PHA II phenotype with an exaggerated response to thiazide diuretics while *Wnk4* ^D561A/+^.*Spak* ^−/−^ mice exhibited a normal phenotype with normal p-NCC expression and response to thiazide. We also created *Wnk4* ^D561A/+^.*Spak* ^−/−^.KSP-*Osr1* ^−/−^ mice, which exhibited low blood pressure with decreased p-Ncc, suggesting that SPAK may plays the more dominant role in the pathogenesis of PHA II but OSR1 can compensate in its absence.

## Materials and Methods

### Animals

The experimental protocols used in the present study were approved by the Institutional Animal Care and Use Committee of the National Defense Medical Center, Taipei, Taiwan. *Wnk4* ^D561A/+^ (C57BL/6 x 129S4/SvJae background), *Spak* ^+/−^ (C57BL/6 and 129X1/SvJ x 129S1 background) and KSP-*Osr1* ^+/−^ (C57BL/6 and 129X1/SvJ x 129S1 background) mice were generated as we described previously.[29], [32], [33] *Wnk4* ^D561A/+^ mice were crossed with *Spak* ^+/−^ and KSP-*Osr1* ^+/−^ to obtain *Wnk4* ^D561A/+^.*Spak* ^+/−^ and *Wnk4* ^D561A/+^.KSP-*Osr1* ^+/−^ mice. Then *Wnk4* ^D561A/+^.*Spak* ^+/−^ and *Wnk4* ^D561A/+^.KSP-*Osr1* ^+/−^ mice were crossed with *Spak* ^+/−^ and KSP-*Osr1* ^+/−^ mice, respectively, to obtain the *Wnk4* ^D561A/+^.*Spak* ^−/−^ and *Wnk4* ^D561A/+^.KSP-*Osr1* ^−/−^ double transgenic mice (Figure S1). *Wnk4* ^D561A/+^.*Spak* ^−/−^.KSP-*Osr1* ^−/−^ triple mutant mice were obtained by systemically breeding *Wnk4* ^+/+^.*Spak* ^+/−^.KSP-*Osr1* ^+/−^ and *Wnk4* ^D561A/+^.KSP-*Osr1* ^+/−^.*Spak* ^+/−^ mice. These mice were raised on a 12-hour day and night cycle and fed normal rodent chow diet [Na^+^: 0.4% (w/w); K^+^: 1.0% (w/w); Ca^2+^: 0.9% (w/w)] and plain drinking water *ad libitum* for 12–14 weeks. The phenotypes of male mice were evaluated at the age of 12–14 weeks.

### Blood and urine analysis and blood pressure measurement

Blood was drawn from the submandibular venous plexus under light ether anesthesia. Mice were kept in metabolic cages for 24-h urine collection. Serum and urine biochemistries and plasma renin activity and aldosterone were measured as previously described.[29], [33], [34] The blood pressure of restrained conscious mice at steady state was measured with a programmable tail-cuff sphygmomanometer (MK-2000A, Muromachi, Tokyo).[34]

### Hydrochlorothiazide (HCTZ) and furosemide administration

Hydrochlorothiazide (HCTZ, 12.5 mg/kg) and furosemide (15 mg/kg) were administered intraperitoneally in *Wnk4* ^D561A/+^.KSP-*Osr1* ^−/−^ and *Wnk4* ^D561A/+^.*Spak* ^−/−^ littermates to determine the activity of Ncc and Nkcc2 *in vivo*. Urine samples were collected for analysis after 4 hours.[29], [33]

### Immunoblotting and immunofluorescence staining

Immunoblotting and immunofluorescence staining were carried out as previously described.[29], [33], [35] The intensities of the resulting immunoblot bands were determined by UVP Bio-imageing system (Cambrige, UK) followed by densitometry (VisionWorksLS Image Acquisition and Analysis Software, Upland, CA). All densitometry data were normalized to the mean of wild type group. The antibodies used in this study include our previously-generated rabbit anti-p-NCC (T53, T58 and S71),[29], [33] anti-p-OSR1(S325)/SPAK(S383)[21], [36] and anti-p-NKCC2 (T96)[29], [33] antibodies, and other commercially available rabbit anti-SPAK (Cell Signaling),[37] Na^+^-K^+^-2Cl^−^ cotransporter 2 (NKCC2) (Alpha Diagnostic),[29], [34] NCC (Millipore),[32] and mouse anti-OSR1 (Abnova).[29], [33] All primary antibodies were used at 1∶200 dilution for immunoblotting. Alkaline phosphatase-conjugated anti-IgG antibodies (1∶3000 dilution, Promega) were used as secondary antibodies for immunoblotting and Alexa 488 or 546 dye-labeled (Molecular Probes) secondary antibodies were used for immunofluorescence staining. The immunofluorescence images were obtained by confocal microscopy (LSM510, Carl Zeiss).

### Statistical analysis

All results are expressed as mean ± standard deviation (SD). The significance of differences between groups was examined by K-independent samples Kruskal-Wallis nonparametric test with SPSS 21.0 for Windows (SPSS, Chicago, IL), followed by Mann-Whitnery two-sample test. The slope of Urine Na^+^ and Cl^−^ excretion rates between before and after diuretics administrating was analyzed by one-sample Kolmogorov-Smirnov test. A *P*-value less than 0.05 was considered to be statistically significant.

## Results

### Phenotypes

We have previously reported the phenotypes of *Wnk4* ^D561A/+^, KSP-*Osr1* ^−/−^, and *Spak* ^−/−^ mice, which recapitulates PHA II, Bartter syndrome and Gitelman syndrome respectively.[29], [33], [34]

### *Wnk4* ^D561A/+^·KSP-*Osr1* ^−/−^ mice

As seen in Table 1, *Wnk4* ^D561A/+^ mice exhibited the typical phenotype of PHA II with significant hypervolemia reflected by higher systolic blood pressure and hyporeninemia, hyperkalemia with decreased fractional urine K^+^ excretion (FE_K_), and hypercalciuria. KSP-*Osr1* ^−/−^ mice exhibited a Bartter syndrome-like phenotype with significant hypokalemia due to excessive renal K^+^ secretion and hypercalciuria. Of note, *Wnk4* ^D561A/+^.KSP-*Osr1* ^−/−^ mice still preserved all the phenotypic indices of PHA II, which were not significantly different from those in *Wnk4* ^D561A/+^ mice, indicating that the renal phenotype of *Wnk4* ^D561A/+^ mice can not be corrected by the genetic deletion of *Osr1* in the kidney.

**Table 1:** Phenotype in *Wnk4* ^D561A/+^·KSP-*Osr1* ^−/−^ littermate mice.

Genotype (*n*)	WT (*6*)	*Wnk4* ^D561A/+^ (*6*)	KSP-*Osr1* ^−/−^ (*6*)	*Wnk4* ^D561A/+^·KSP-*Osr1* ^−/−^ (*6*)
**Systolic BP** (mmHg)	112.5±10	135.3±9.1^a^	108.0±8.1^b^	133.6±11^a,c^
**Body Weight** (g)	29.7±5.1	26.1±4.5	26.1±4.6	25.6±1.5
**Plasma**				
Aldosterone (pg/ml)	832±120.0	850±250.0	798±88.5	819±144.1
PRA(ng ml h)	9.0±5.4	5.5±1.6^a^	10.2±3.1^b^	4.2±2.2^a,c^
Na^+^ (mmol/l)	152±3	151±4	154±3	153±4
K^+^ (mmol/l)	4.2±0.3	4.9±0.2^a^	3.9±0.2^a,b^	4.8±0.2^a,c^
Cl^−^ (mmol/l)	115±2	118±2^a^	114±3	119±2^a,c^
HCO_3_^−^ (mmol/l)	26±4.0	22±2.5^a^	27±5.1^b^	23±3.1^a,c^
Total Ca^2+^ (mg/dl)	9.5±0.1	9.5±0.3	9.6±0.3	9.4±0.2
Mg^2+^ (mg/dl)	2.5±0.1	2.4±0.5	2.6±0.2	2.6±0.3
Cr (mg/dl)	0.20±0.08	0.19±0.02	0.20±0.07	0.24±0.02
**Urine** (ml/day)	2.2±0.5	2.3±0.4	2.7±0.6^a,b^	2.3±0.3^c^
FE_Na_ (%)	0.26±0.06	0.29±0.08	0.27±0.07	0.31±0.10
FE_K_ (%)	15.5±3.0	10.0±3.5^a^	19.3±2.8^a,b^	12.0±2.0^a,c^
FE_Cl_ (%)	0.25±0.04	0.26±0.06	0.28±0.10	0.26±0.13
FE_Mg_(%)	14.2±2.7	14.1±3.5	13.8±2.5	14.2±3.4
Ca^2+^/Cr (mg/mg)	0.21±0.03	0.35±0.02^a^	0.32±0.04^a^	0.33±0.03^a^

BP, blood pressure; PRA, plasma rennin activity; Cr, creatinine; FE_Na_, FE_K,_ FE_Cl_ and FE_Mg_ represent the fractional excretion of Na^+^, K^+^, Cl^−^ and Mg^2+^ respectively. **^a^** *p*<0.05 vs. WT. **^b^** *p*<0.05 vs. *Wnk4* ^D561A/+^. **^c^** *p*<0.05 vs. KSP-*Osr1* ^−/−^.

### *Wnk4* ^D561A/+^·*Spak* ^−/−^ mice

*Spak* ^−*/*−^ mice resembled the phenotype of Gitelman syndrome with relative hypotension, secondary hyperaldosteronism, low plasma K^+^ concentration with increased FE_K_, hypomagnesemia with increased fractional urine Mg^2+^ excretion (FE_Mg_), and hypocalciuria (Table 2). *Wnk4* ^D561A/+^.*Spak* ^−/−^ mice became normotensive and exhibited similar plasma and urine indices as WT mice (except fractional urine Cl^−^ excretion) suggesting that the phenotype of *Wnk4* ^D561A/+^ mice can be corrected by *Spak* deficiency in the kidney.

**Table 2:** Phenotype in *Wnk4* ^D561A/+^·*Spak* ^−/−^ littermate mice.

Genotype (*n*)	WT (*7*)	*Wnk4* ^D561A/+^ (*7*)	*Spak* ^−/−^ (*7*)	*Wnk4* ^D561A/+^·*Spak* ^−/−^ (*7*)
**Systolic BP** (mmHg)	109.8±2.9	128.3±2.2^a^	101.4±4.0^a,b^	107.6±4.6^b,c^
**Body Weight** (g)	21.3±3.2	23.9±3.1	24.4±4.4	22.4±4.3
**Plasma**				
Aldosterone (pg/ml)	831±262.7	910±322	1430±301.4^a,b^	820±234^c^
PRA(ng·ml·h)	5.5±1.0	2.2±0.4^a^	8.9±1.5^a,b^	5.1±2.1^b,c^
Na^+^ (mmol/l)	149±5	152±2	148±3	151±2
K^+^ (mmol/l)	4.2±0.2	4.9±0.2^a^	3.6±0.2^a,b^	4.3±0.5^b,c^
Cl^−^ (mmol/l)	114±2	117±3^a^	109±2^a,b^	113±3^b,c^
HCO_3_^−^ (mmol/l)	25±5	21±2^a^	30±4^a,b^	24±3^b,c^
Total Ca^2+^ (mg/dl)	9.6±0.2	9.5±0.3	9.4±0.1	9.5±0.4
Mg^2+^ (mg/dl)	2.4±0.2	2.3±0.5	1.9±0.3^a,b^	2.4±0.5
Cr (mg/dl)	0.18±0.07	0.17±0.06	0.19±0.05	0.18±0.08
**Urine **(ml/day)	2.26±0.8	2.32±0.5	2.20±0.6	2.23±0.9
FE_Na_ (%)	0.26±0.05	0.27±0.08	0.25±0.12	0.28±0.09
FE_K_ (%)	16.4±1.7	13.0±2.1^a^	21.0±1.0^a,b^	17.0±5.1^b,c^
FE_Cl_ (%)	0.27±0.15	0.28±0.14	0.29±0.20	0.26±0.18
FE_Mg_(%)	12.6±1.8	13.5±2.8	16.4±1.6^a,b^	12.7±3.2^c^
Ca^2+^/Cr (mg/mg)	0.25±0.04	0.36±0.08^a^	0.16±0.01^a,b^	0.23±0.05^b,c^

BP, blood pressure; PRA, plasma rennin activity; Cr, creatinine; FE_Na_, FE_K,_ FE_Cl_ and FE_Mg_ represent the fractional excretion of Na^+^, K^+^, Cl^−^ and Mg^2+^ respectively. **^a^** *p*<0.05 vs. WT. **^b^** *p*<0.05 vs. *Wnk4* ^D561A/+^. **^c^** *p*<0.05 vs. *Spak* ^−/−^.

### *Wnk4* ^D561A/+^·KSP-*Osr1* ^−/−^·*Spak* ^−/−^ mice

Out of 160 offsprings, we only obtained two *Wnk4* ^D561A/+^.KSP-*Osr1* ^−/−^.*Spak* ^−/−^ mice (n = 2). These mice exhibited relative hypotension (97± 5.0 mmHg vs 110± 4.0 mmHg in WT) and mild hypokalemia (3.7±0.3 mmol/l vs 4.2±0.4 mmol/l in WT ) as compared to their WT littermates.

### Protein expression and phosphorylation of Ncc, Nkcc2, Spak, and Osr1

**Figure 1:**
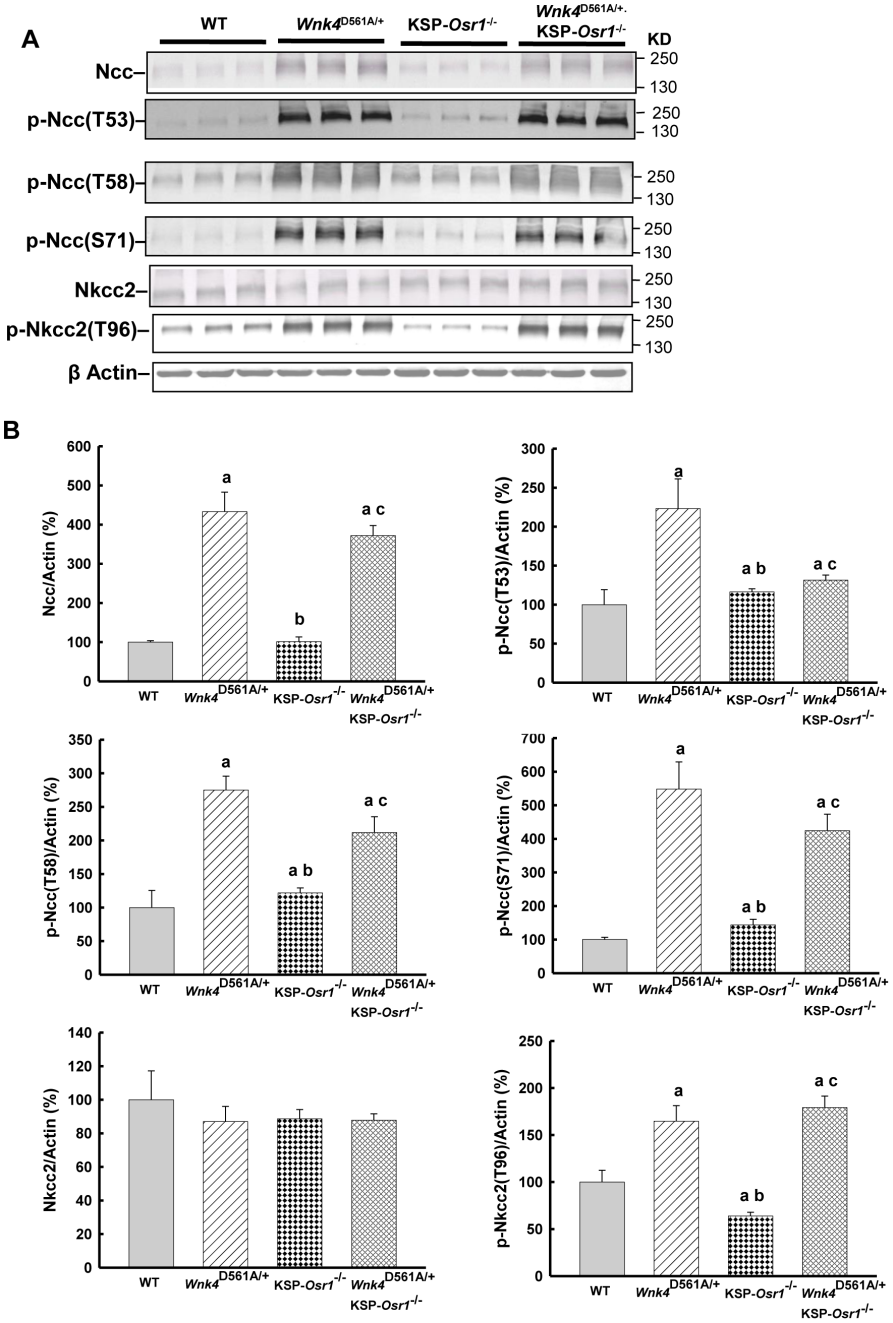
*Osr1* gene deletion does not change phosphorylation of Ncc and Nkcc2 in PHA II. Shown is the representative immunoblot (top, n = 3/group) and densitometry (bottom, n = 6/group) of (A) total Ncc, (B) p-Ncc (T53), (C) p-Ncc (T58), (D) p-Ncc (S71), (E) total Nkcc2, and (F) p-Nkcc2 (T96) in whole kidneys of WT, *Wnk4* ^D561A/+^, KSP-*Osr1* ^−/−^, and *Wnk4* ^D561A/+^.KSP-*Osr1* ^−/−^ mice, representively. Semiquantitative measurements of each band in the gel were performed and the results are shown as mean ± SD. **^a^** *p*<0.05 vs. WT. **^b^** *p*<0.05 vs. *Wnk4* ^D561A/+^. **^c^** *p*<0.05 vs. KSP-*Osr1* ^−/−^.

**Figure 2:**
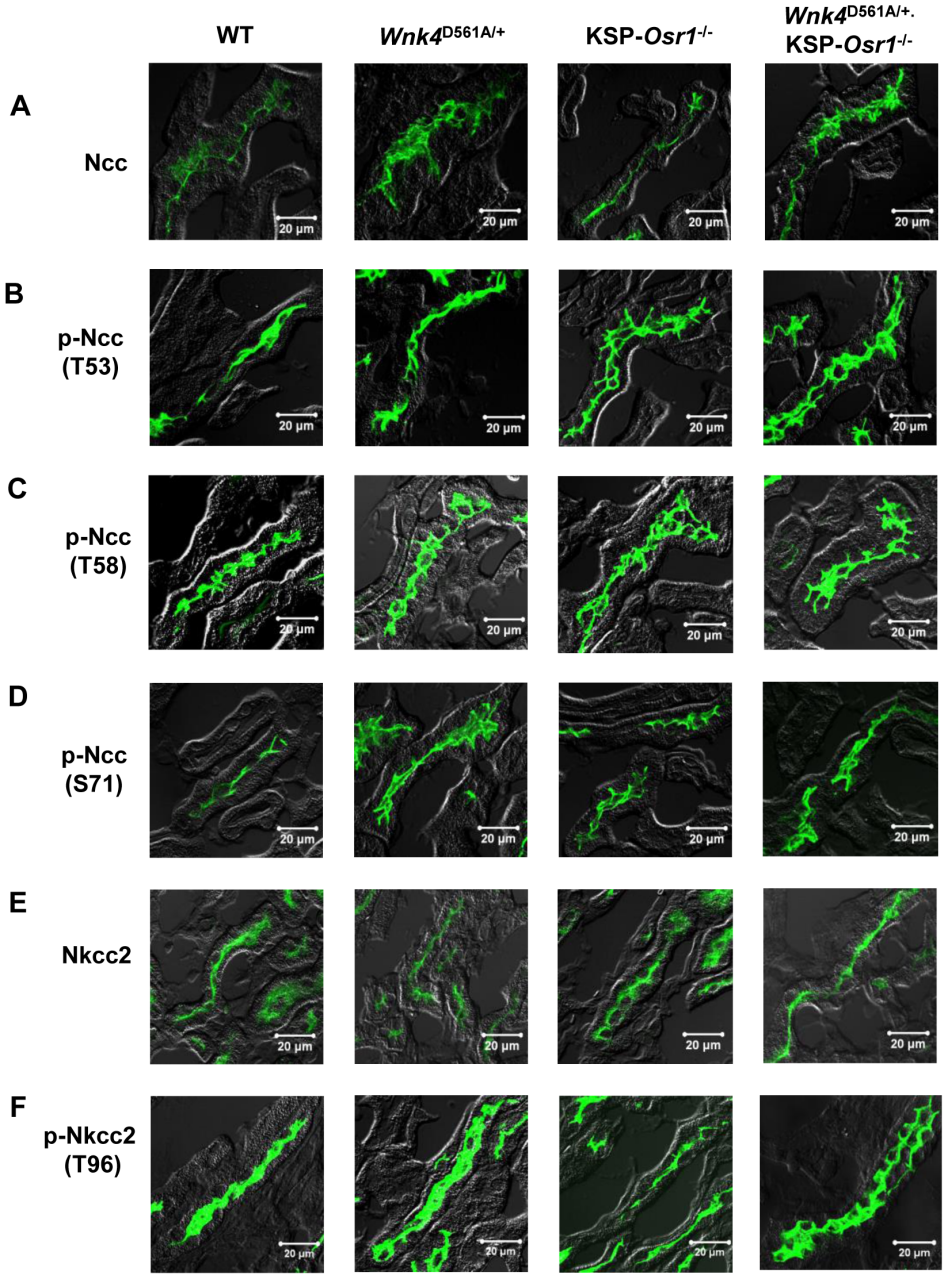
Immunofluorescence images of Ncc and Nkcc2 in kidneys. (Osr1 experiment series) Representative immunofluorence images of (A) total Ncc, (B) p-Ncc (T53), (C) p-Ncc (T58), (D) p-Ncc (S71), (E) total Nkcc2, and (F) p-Nkcc2 (T96) in kidneys of WT, *Wnk4* ^D561A/+^, KSP-*Osr1* ^−/−^, and *Wnk4* ^D561A/+^.KSP-*Osr1* ^−/−^ mice. The scale bars indicate 20 µm.

Compared to WT controls, *Wnk4* ^D561A/+^ mice had increased expressions of total Ncc (433.0±49.7%, p<0.01), p-NccT53 (223.2±38.1%, p<0.01), p-NccT58 (275.0±20.7%, p<0.01), p-NccS71 (548.3±80.8%, p<0.01) (Figure 1A-D), and p-Nkcc2 (164.6±16.6%, p<0.01, Figure 1F) assessed by semi-quantitative immunoblotting of whole kidney homogenate. As shown in Figure S2, Nkcc2 was predominantly expressed in the medulla and p-Nkcc2 mostly expressed in the cortex of WT mice. In *Wnk4* ^D561A/+^ mice, the abundance of Nkcc2 in the medullar region was reduced but p-Nkcc2 in the cortical region was increased. KSP*-Osr1* ^−/−^ mice had slightly increased total Ncc (101.4±12.0%, Figure 1A) and p-NccT53, T58 and S71 (116.4±4.0%, 121.8±7.5%, 143.9±16.5%, all p<0.05, Figure 1B-D), but a significant decreased p-Nkcc2 (63.9±4.0%, p<0.01, Figure 1F) with unchanged total Nkcc2 (Figure 1E). *Wnk4* ^D561A/+^.KSP-*Osr1* ^−/−^ mice still had significantly increased total Ncc (371.9±25.8%, p<0.01), p-NccT53 (131.4±6.6%, p<0.01, Figure 1B), p-NccT58 (211.8±23.6%, p<0.01), and p-NccS71 (424.2±49.4%, p<0.01) (Figure 1A-D), and p-Nkcc2 (179.2±12.3%, p<0.01, Figure 1F) with unchanged Nkcc2. Similar to *Wnk4* ^D561A/+^ mice, the expression of p-Spak was increased in KSP-*Osr1* ^−/−^ and *Wnk4* ^D561A/+^.KSP-*Osr1* ^−/−^ mice (data not shown). The results measured by semi-quantitative immunofluorescence of Ncc and Nkcc2 in the kidney sections of WT, *Wnk4* ^D561A/+^, KSP-*Osr1* ^−/−^, and *Wnk4* ^D561A/+^.KSP-*Osr1* ^−/−^ mice were consistent with the immunoblotting findings (Figure 2 and Figure S3).

**Figure 3:**
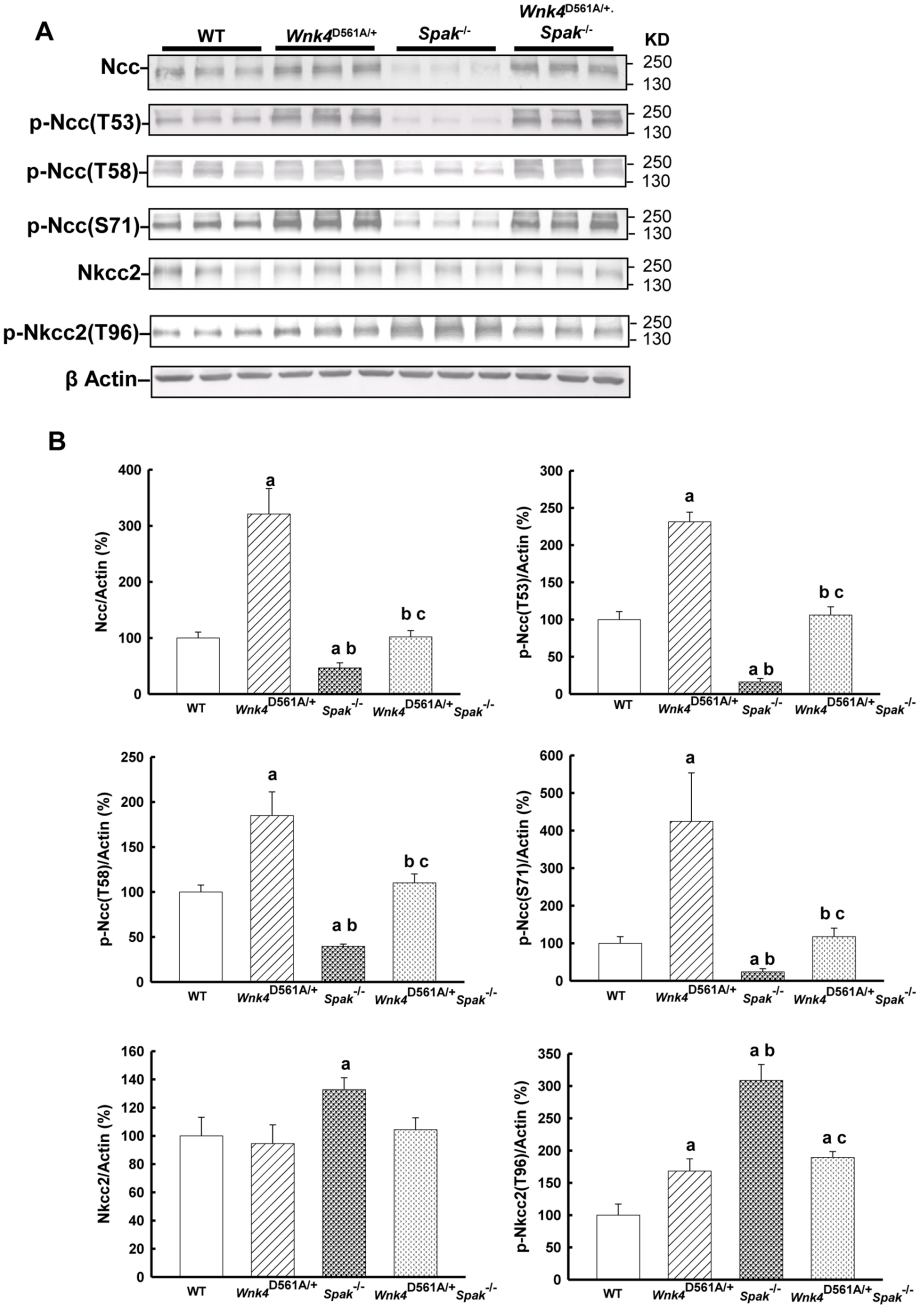
*Spak* gene deletion normalizes Ncc phosphorylation but not Nkcc2 phosphorylation in PHA II. Shown is the representative immunoblot (top, n = 3/group) and densitometry (bottom, n = 6/group) of (A) total Ncc, (B) p-Ncc (T53), (C) p-Ncc (T58), (D) p-Ncc (S71), (E) total Nkcc2, and (F) p-Nkcc2 (T96) in the whole kidneys of WT, *Wnk4* ^D561A/+^, *Spak* ^−/−^, and *Wnk4* ^D561A/+^.*Spak* ^−/−^ mice, representively. The results are shown as mean ± SD. **^a^** *p*<0.05 vs. WT. **^b^** *p*<0.05 vs. *Wnk4* ^D561A/+^. **^c^** *p*<0.05 vs. *Spak* ^−/−^.

**Figure 4:**
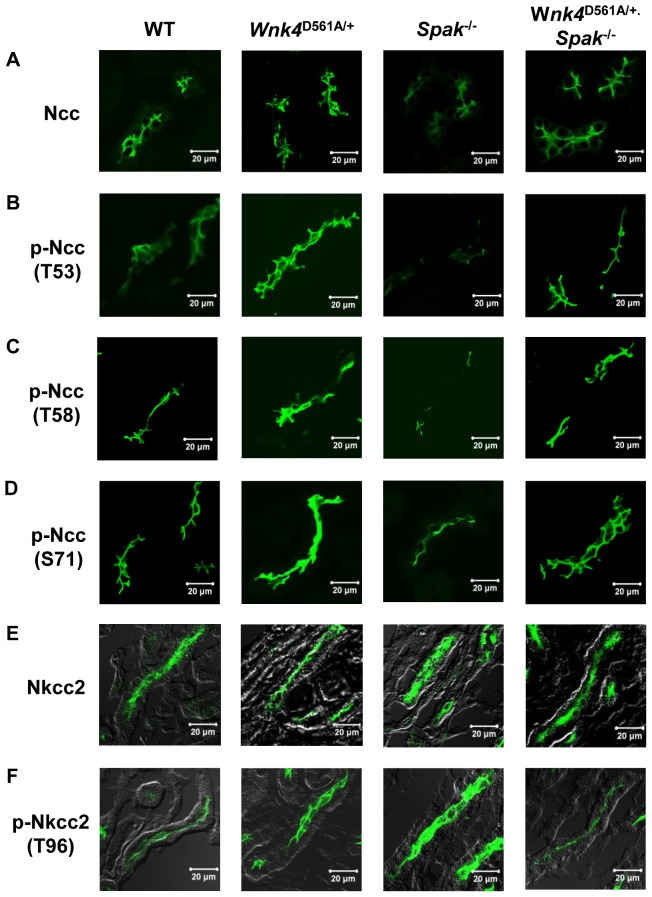
Immunofluorescence images of Ncc and Nkcc2 in kidneys. (Spak experiment series ) Representative immunofluorescence image of (A) total Ncc, (B) p-Ncc (T53), (C) p-Ncc (T58), (D) p-Ncc (S71), (E) total Nkcc2, and (F) p-Nkcc2 (T96) in kidneys of WT, *Wnk4* ^D561A/+^, *Spak* ^−/−^, and *Wnk4* ^D561A/+^.*Spak* ^−/−^ mice. The scale bars indicate 20 µm.

In *Spak* ^−/−^ mice, the expression of total Ncc (46.7±9.0%, p<0.01) and p-NccT53 (16.2±4.7%, p<0.01), p-NccT58 (39.5±2.6%, p<0.01) and p-NccS71 (23.8± 8.7%, p<0.01) (Figure 3A-D) were markedly attenuated but total Nkcc2 (132.8±.4%, p<0.01, Figure 3E) and p-Nkcc2 (308.6±24.6%, p<0.01, Figure 3F) were significantly increased. Despite increased p-Osr1 (data not shown), *Wnk4* ^D561A/+^.*Spak* ^−/−^ mice had similar expressions of total Ncc, p-Ncc, total Nkcc2, p-Nkcc2 to wild type (Figure 3A-F). The immunofluoresence results of total and phosphorylated Ncc and Nkcc2 expression in the kidney sections of WT, *Wnk4* ^D561A/+^, *Spak* ^−/−^, and *Wnk4* ^D561A/+^.*Spak* ^−/−^ mice (Figure 4 and Figure S4) were also consistent with those immunoblotting observations.

**Figure 5:**
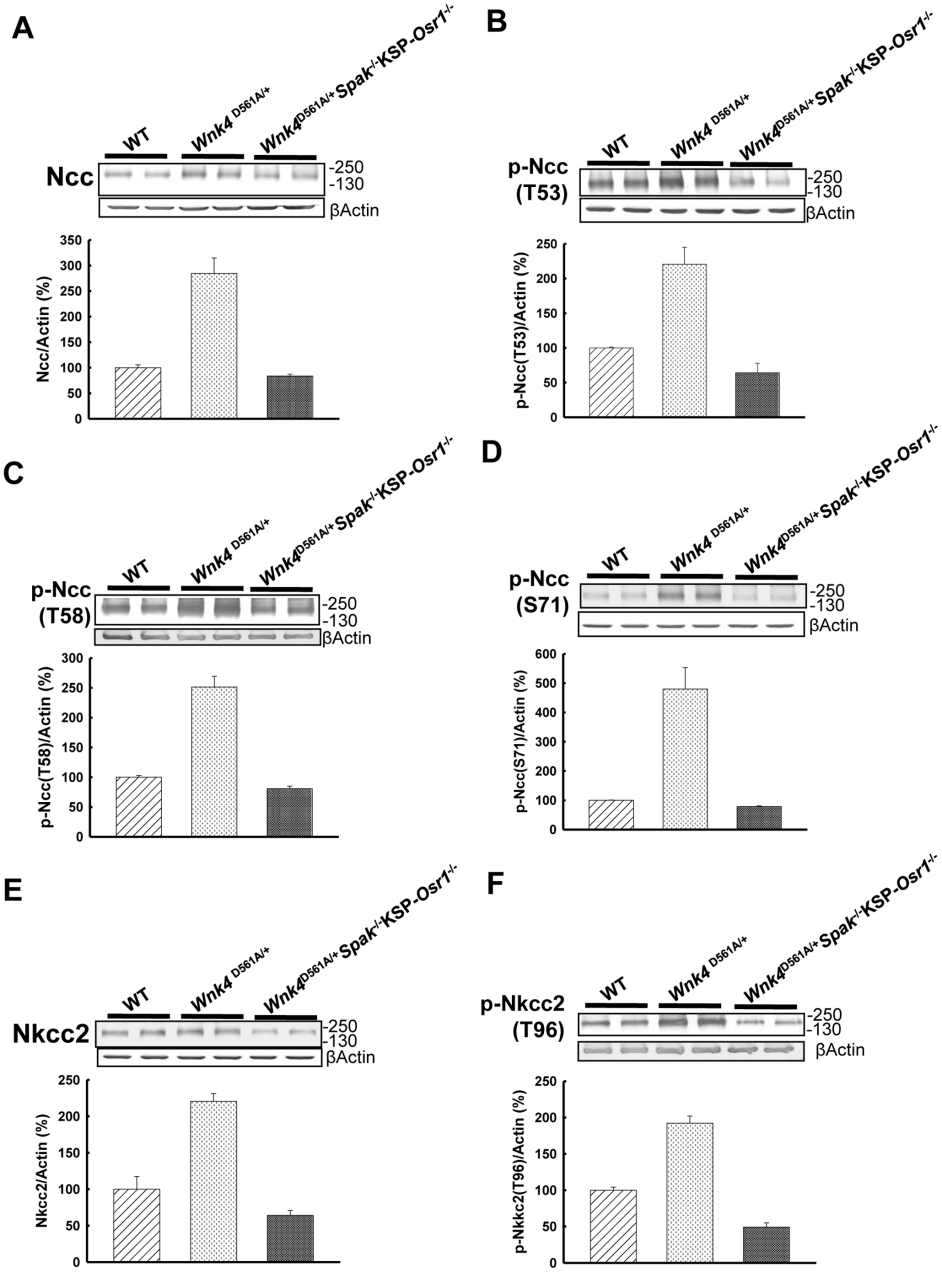
Double deletion of *Spak* and *Osr1* genes decreased Ncc phosphorylation and Nkcc2 phosphorylation in *Wnk4*-mutant PHA II. Shown is the representative immunoblot (top) and densitometry (bottom) of (A) total Ncc, (B) p-Ncc (T53), (C) p-Ncc (T58), (D) p-Ncc (S71), (E) total Nkcc2, and (F) p-Nkcc2 (T96) in the kidneys of WT (1), *Wnk4* ^D561A/+^ (2), *Wnk4* ^D561A/+^ *Spak* ^−/−^KSP-*Osr1* ^−/−^ (3) mice, respectively. The results are shown as mean ± SD (n = 2/group).

In triple *Wnk4* ^D561A/+^.*Spak* ^−/−^.KSP-*Osr1* ^−/−^mice (n = 2/each group), the expression of total Ncc (83.5±1.9%) and p-NccT53 (64.1±13.8%), p-NccT58 (80.9±1.2%) and p-NccS71 (78.2±1.9%) were markedly decreased compared to *Wnk4* ^D561A/+^ mice (Figure 5A-D). Total Nkcc2 (76.6±6.7%) (Figure 5E) and p-Nkcc2 (49.2±5.7%) (Figure 5F) were also decreased in *Wnk4* ^D561A/+^.*Spak* ^−/−^.KSP-*Osr1* ^−/−^ triple mutant mice than WT and *Wnk4* ^D561A/+^ mice (Nkcc2: 87.3±10.8%; p-Nkcc2: 192.2±9.8%, Figure 5E-F).

### Response to diuretics

**Figure 6:**
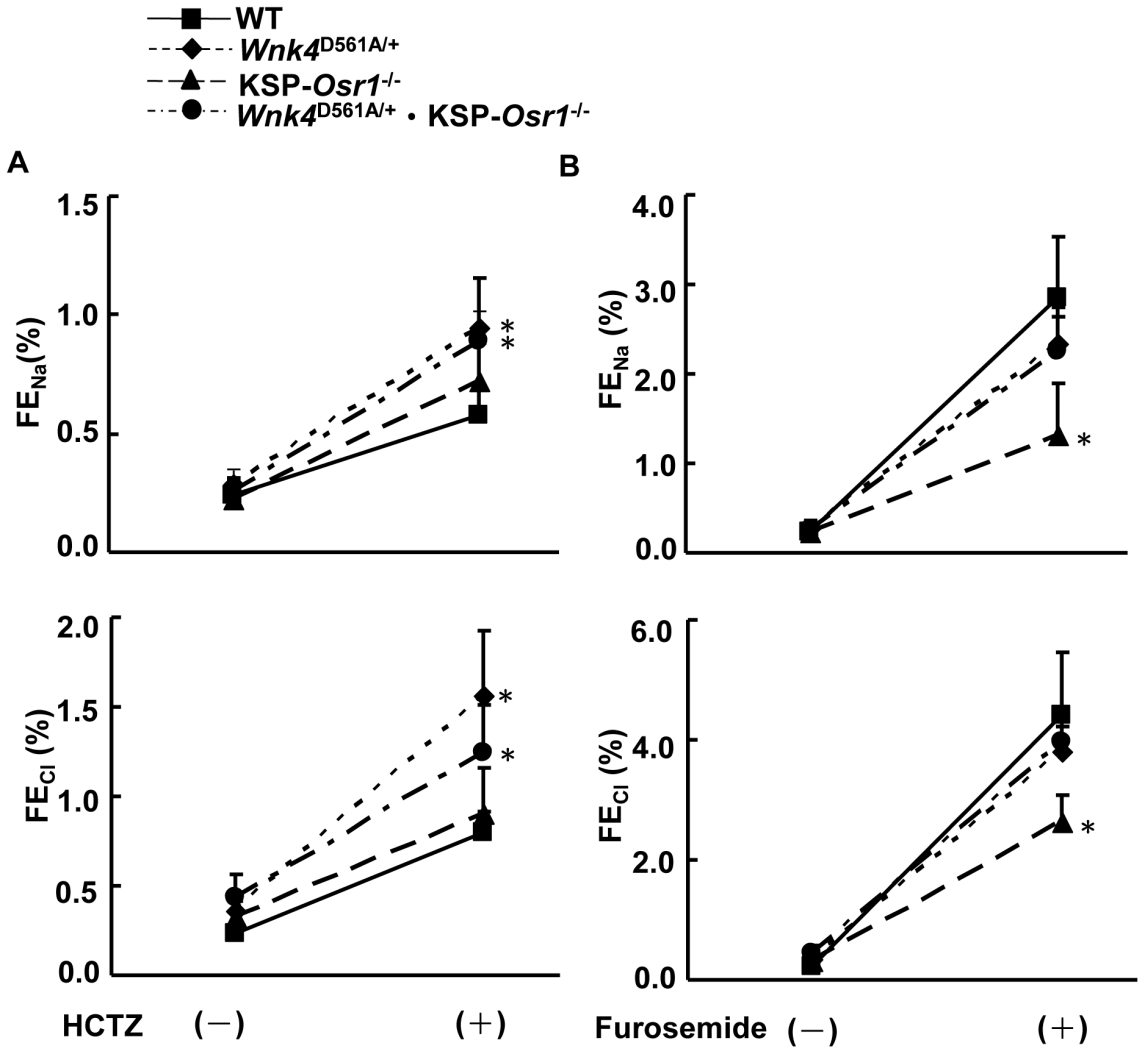
*Osr1* gene deletion does not change the response to hydrochlorothiazide (HCTZ) and furosemide in PHA II mice. FE_Na_ and FE_Cl_ represent the fractional excretion of Na^+^ and Cl^−^ respectively. Responses of FE_Na_ and FE_Cl_ in WT (▪), *Wnk4* ^D561A/+^ (♦), KSP-*Osr1* ^−/−^ (▴), and *Wnk4* ^D561A/+^.KSP-*Osr1* ^−/−^ (•) littermate mice (n = 6/group) to (A) HCTZ and (B) furosemide. *p<0.05 vs. WT.

Urine Na^+^ and Cl^−^ excretion rates (FE_Na_ and FE_Cl_) were measured in mice before and after the administration of hydrochlorothiazide (HCTZ, a Ncc inhibitor) or furosemide (a Nkcc2 inhibitor) to assess the *in vivo* activities of Ncc and Nkcc2 respectively. Compared with WT and KSP-*Osr1* ^−/−^ mice, *Wnk4* ^D561A/+^ and *Wnk4* ^D561A/+^.KSP-*Osr1* ^−/−^ mice exhibited exaggerated salt excretion in response to a single dose of HCTZ, indicating the Ncc overactivity in both sets of mice (Figure 6A). When challenged with furosemide, KSP-*Osr1* ^−/−^ mice showed blunted response in comparison with WT, suggesting lower Nkcc2 activity (Figure 6B). However, *Wnk4* ^D561A/+^ and *Wnk4* ^D561A/+^.KSP-*Osr1* ^−/−^ mice responded similarly to WT.

**Figure 7:**
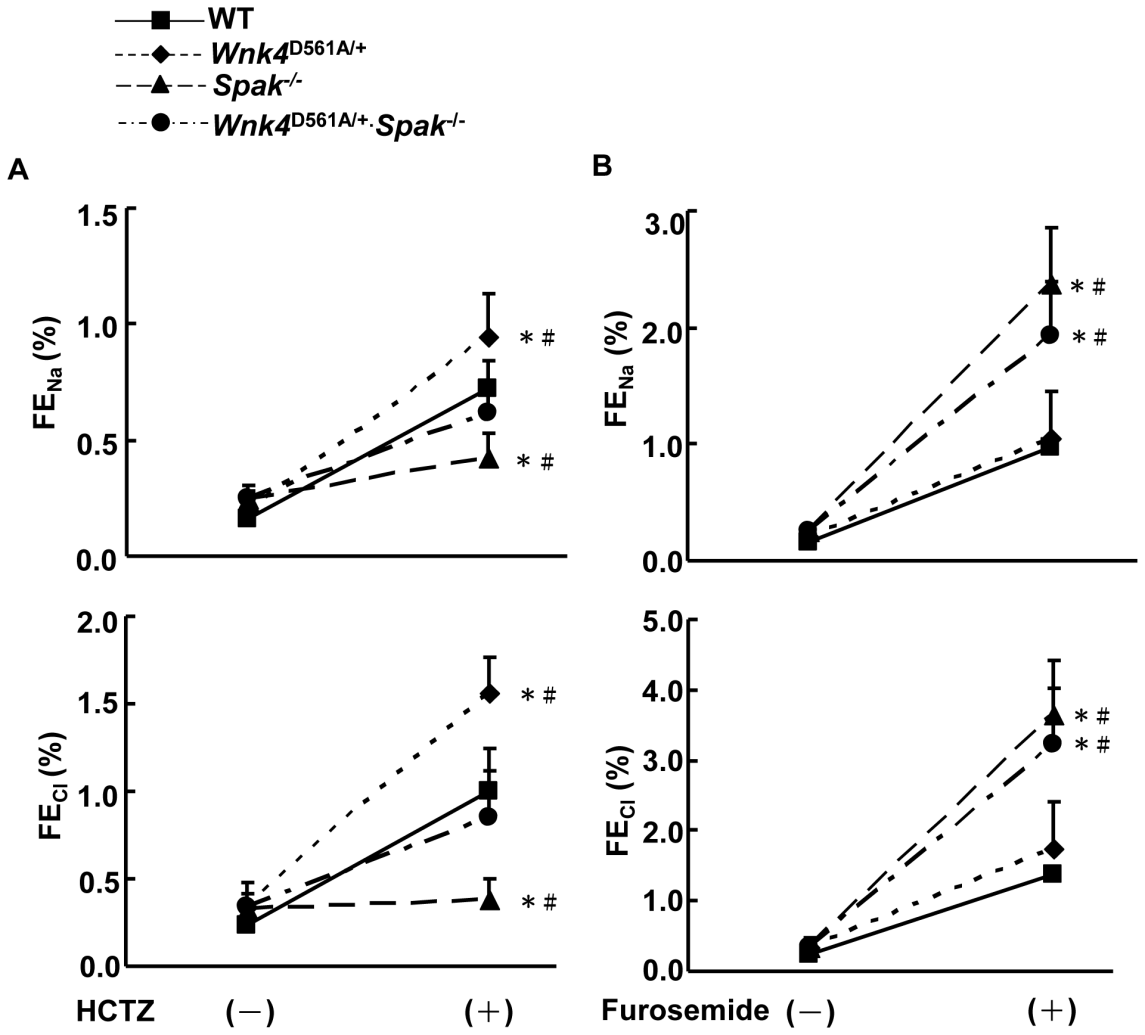
*Spak* gene deletion normalizes the response to HCTZ and exaggerates the response to furosemide in PHA II mice. Responses of FE_Na_ and FE_Cl_ in WT (▪), *Wnk4* ^D561A/+^ (♦), *Spak* ^−/−^ (▴), and *Wnk4* ^D561A/+^.*Spak* ^−/−^ (•) littermate mice (n = 6/group) to (A) HCTZ and to (B) furosemide. *denotes *p*<0.05 vs. WT. ^#^ *p*<0.05 vs. *Wnk4* ^D561A/+^.

The *Spak* ^−/−^ mice had blunted urine Na^+^ and K^+^ excretion compared to WT in response to HCTZ (Figure 7A), indicating lower Ncc activity. Interestingly, the response of *Wnk4* ^D561A/+^.*Spak* ^−/−^ mice was between that of *Spak* ^−/−^ and *Wnk4* ^D561A/+^ mice and similar to WT controls, indicating that the Ncc function had normalized in these mice. In the furosemide challenge, *Spak* ^−*/*−^ and *Wnk4* ^D561A/+^.*Spak* ^−/−^ mice had significantly increased Na^+^ and Cl^−^ excretion, suggesting increased Nkcc2 function (Figure 7B).

## Discussion

In this study, we crossed *Wnk4* ^D561A/+^ mice with KSP-*Osr1* ^−/−^ and *Spak* ^−/−^ mice to investigate the independent roles of Osr1 and Spak in the pathogenesis of *Wnk4-*PHA II. *Wnk4* ^D561A/+^.KSP-*Osr1* ^−/−^ mice preserved the PHA II phenotype with increased abundance of p-Spak and p-Ncc and corresponding exaggerated response to thiazide diuretics. *Wnk4* ^D561A/+^.*Spak* ^−/−^ mice exhibited normal blood pressure and blood/urine electrolytes with relatively normal abundance of p-Ncc despite enhanced p-Osr1 expression and a normal response to thiazides. These findings indicated that activation of Spak-Ncc plays the more dominant role in *Wnk4*-PHA II, which is affirmed by the decreased total expresssion and phosphorylation of Ncc in triple mutant *Wnk4* ^D561A/+^.*Spak* ^−/−^.KSP-*Osr1* ^−/−^ mice.

Mutations in the *Wnk4* kinase gene have been shown to cause many cases of PHA II.[15], [38]–[40] Recent studies have implicated the activation of downstream WNK4 substrates, SPAK and OSR1, in the pathogenesis of PHA II.[26], [32], [36], [41], [42] Although SPAK has been found to be predominantly expressed in the cortex and OSR1 in the medulla, both are expressed in the TAL and DCT.[29] Unlike the interchangeability of SPAK and OSR1 in peripheral neurons,[43] these two kinases seem to be differentially regulated and have different function in renal tubules. This study clarified the relative contribution of Spak and Osr1 to PHA II *in vivo*. *Wnk4* ^D561A/+^.KSP-*Osr1* ^−/−^ mice had increased expression of total Ncc and p-Ncc in parallel with increased total and p-Spak. Their exaggerated response to thiazide diuretics indicated Ncc hyperactivity, similar to *Wnk4* ^D561A/+^mice, and in line with the immunoblottings. These findings suggest that Osr1 is not essential and can be fully compensated by the increased Spak expression in this modle of PHA II. However, the decreased p-Ncc expression found in heterozygous kinase-dead *Osr1* knockin (*Osr1* ^T158A/+^) mice and *Wnk4* ^D561A/+^.KSP-*Osr1* ^−/−^.*Spak* ^−/−^ triple mutant mice suggested that the rescue of Ncc activation in Osr1 deficient states depends on abnormal activation of the Spak pathway.[41]

The roles of Osr1 and Spak on Nkcc2 in TAL were also clarified by this study. KSP-*Osr1* ^−/−^ mice exhibited reduced p-Nkcc2, indicating that Osr1 is an up-regulator of Nkcc2. Substantively, the *Spak* ^−/−^, *Wnk4* ^D561A/+^, and *Wnk4* ^D561A/+^.*Spak* ^−/−^ mice all exhibited increased p-Nkcc2. These three sets of mice share the commonality of preserved or increased Osr1. However, the *Wnk4* ^D561A/+^.KSP-*Osr1* ^−/−^ mice also exhibited increased p-Nkcc2 suggesting that mutant *Wnk4* can activate Nkcc2 through activited Spak. This is corrobarated by our finding of decreased p-Nkcc2 when Spak is abolished in the triple mutant *Wnk4* ^D561A/+^.KSP-*Osr1* ^−/−^.*Spak* ^−/−^ mice. It would appear that Osr1 is the major activator of Nkcc2 but Spak may play a role in abnormally activated states. Recently, it has reported that the kinase-deficient SPAK variant, so-called kidney specific SPAK (KS-SPAK), functions as an antagonist of OSR/SPAK-NKCC2 pathway and is the major SPAK isoform in renal medulla.[27] Since WNK4 expression in the Henle’s loop is primarily in the cortical TAL,[44] the role of KS-SPAK in PHA II with WNK 4 mutation is still questionable.

The furosemide challenge studies generally correlate with the densitometry studies except in the *Wnk4* ^D561A/+^ and *Wnk4* ^D561A/+^.KSP-*Osr1* ^−/−^ mice, which had normal responses to furosemide despite increased phosphorylated Nkcc2. It is important to note that both of these mice have hyperactive downstream Ncc, which may attenuate the observable response to furosemide. Supporting this theory is the observation that *Wnk4* ^D561A/+^.*Spak* ^−/−^ mice, with their increased activated Osr1 and p-Nkcc2 but relatively normal p-Ncc expression, showed an exaggerated response to furosemide, providing direct evidence linking increased Nkcc2 activity through Osr1. Besides Wnk4, other upstream regulator, such as WNK1 or calcium-binding protein 39 (Cab39), also regulate OSR1-NKCC2 pathway.[19], [21], [45]–[47].

Regarding the Ncc in the distal nephron, Spak appears to be the dominant player as *Wnk4* ^D561A/+^.*Spak* ^−/−^ mice became virtually normal phenotype with expression levels of total and p-Ncc similar to WT littermates, indicating that the PHA II phenotype could be effectively corrected by *Spak* deficiency. The response to thiazide diuretics in *Wnk4* ^D561A/+^.*Spak* ^−/−^ mice was similar to WT mice (and higher than *Spak* ^−/−^ mice) suggesting that Spak is a major but not sole activator of Ncc in *Wnk4*-PHA II. Osr1 is a likely accomplice and increased Osr1 activity through activated Wnk4 may compensate enough to sustain normal Ncc expression and activity. This borne out by the finding of decreased expression and phosphorylation of Ncc in triple mutant *Wnk4* ^D561A/+^.KSP-*Osr1* ^−/−^.*Spak* ^−/−^ mice, where Osr1 has been abolished. The phenotype and Ncc phosphorylation level of our *Wnk4* ^D561A/+^.*Spak* ^−/−^ mice resembled those of the recently-reported *Wnk4* ^D561A/+^.*Spak* ^T243A/T243A^ mice (kinase-dead knock-in),[41] which also support the importance of SPAK kinase activity in PHA II. Another recent study has demonstrated that WNK4-SPAK-dependent signaling is the primary mechanism behind angiotensin II induced Ncc stimulation.[42] The WNK4-NCC signaling pathway is also regulated by other hormones (aldosterone and insulin) and drugs (tacrolimus, cyclosporine) associated with salt-sensitive hypertension.[36], [48]–[51] Whether those mechanisms are principally mediated through SPAK warrants further investigation.

Historically, WNK4 was reported to inhibit membrane trafficking of NCC based on oocyte experiments.[52]–[54] However, this Ncc inhibitory mechanism has not been found *in vivo*. Similarly, *in vitro* studies proposing various mechanisms WNK4-related for Ncc degradation are equally suspect.[55], [56] How WNK4 directly affects NCC *in vivo* merits further study.

Thiazide diuretics are commonly and effectively used to treat human PHA II disease. However, the chronic use of thiazide also cause several side effects, such as insulin resistance with hyperglycemia, hyperlipidemia, hyperuricemia with gout, chronic kidney injury and even renal failure. These side effects can be independent of volume status and plasma K^+^ concentration.[57] Because both *Spak* deficiency and inhibition of Spak kinase activity corrected the phenotype of PHA II due to *Wnk4* mutation, specific inhibition of SPAK may be a plausible therapy for patients with salt-sensitive hypertension related to WNK4 activation. Since human PHA II is also linked to the mutations in WNK1, Kelch-like 3 or cullin 3 genes, the SPAK in those gene mutations will need to be clarified first.[16], [17], [20], [58]–[60].

In conclusion, *Wnk4* ^D561A/+^.KSP-*Osr1* ^−/−^double transgenic mice preserved the PHA II phenotype with evidences of Ncc overactivity while *Wnk4* ^D561A/+^.*Spak* ^−/−^ mice exhibited normal phenotype with relatively normal Ncc activity. Spak appears to be the dominant activator of Ncc while Osr1 is the major activator of Nkcc2. However, they may be overdriven to compensate for the other’s absence in PHA II conditions. Disruption of Spak-Ncc cascade can efficiently correct hypertension and hyperkalemia in the Wnk4-PHA II mouse model. This study suggests that inhibition of SPAK can be a promising therapy for salt-sensitive hypertension with WNK4-SPAK-dependent NCC activation.

## Supporting Information

Figure S1 Expression of Osr1 and Spak in kidneys of *Wnk4* ^D561A/+^.KSP-*Osr1* ^−/−^ and *Wnk4* ^D561A/+^.*Spak* ^−/−^ mice. Representative immunoblots from three separate experiments of (A) Osr1 (top) and phosphorylated (p-)Osr1 (bottom) abundance in the kidneys of WT, *Wnk4* ^D561A/+^, KSP-*Osr1* ^−/−^, and *Wnk4* ^D561A/+^.KSP-*Osr1* ^−/−^ mice and (B) Spak (top) and p-Spak (bottom) abundance in the kidneys of WT, *Wnk4* ^D561A/+^, *Spak* ^−/−^, and *Wnk4* ^D561A/+^.*Spak* ^−/−^ mice.
(PPT)

Figure S2 Immunofluorescence images of Nkcc2 and p-Nkcc2(T96) in kidneys of WT and *Wnk4* ^D561A/+^mice. In WT mice, Nkcc2 (red) was dominantly expressed in the medullar (M) region and p-Nkcc2 (green) was mostly expressed in the cortical (C) region. In *Wnk4* ^D561A/+^ mice, the abundance of Nkcc2 (red) in the medullar region was reduced but p-Nkcc2(green) in the cortical region was enhanced. The scale bars indicate 100 µm.
(PPT)

Figure S3 Low-power immunofluorescence of renal Ncc and Nkcc2. (Osr1 experiment series) Representative (A) total Ncc, (B) p-Ncc (T53), (C) p-Ncc (T58), (D) p-Ncc (S71), (E) total Nkcc2, and (F) p-Nkcc2 (T96) in kidneys of WT, *Wnk4* ^D561A/+^, KSP-*Osr1* ^−/−^, and *Wnk4* ^D561A/+^.KSP-*Osr1* ^−/−^ mice. The scale bars indicate 100 µm.
(PPT)

Figure S4 Low-power immunofluorescence of renal Ncc and Nkcc2. (Spak experiment series) Representative (A) total Ncc, (B) p-Ncc (T53), (C) p-Ncc (T58), (D) p-Ncc (S71), (E) total Nkcc2, and (F) p-Nkcc2 (T96) in kidneys of WT, *Wnk4* ^D561A/+^, *Spak* ^−/−^, and *Wnk4* ^D561A/+^.*Spak* ^−/−^ mice. The scale bars indicate 100 µm.
(PPT)
