# Supplementary material for: SPAK Deficiency Corrects Pseudohypoaldosteronism II Caused by WNK4 Mutation
Source: PLoS One. 2013 Sep 11;8(9):e72969. doi: 10.1371/journal.pone.0072969 (PMC3770638; doi:10.1371/journal.pone.0072969)
Supplement: Figure S1 — Expression of Osr1 and Spak in kidneys of Wnk4 D561A/+.KSP-Osr1 −/− and Wnk4 D561A/+.Spak −/− mice. Representative immunoblots from three separate experiments of (A) Osr1 (top) and phosphorylated (p-)Osr1 (bottom) abundance in the kidneys of WT, Wnk4 D561A/+, KSP-Osr1 −/−, and Wnk4 D561A/+.KSP-Osr1 −/− mice and (B) Spak (top) and p-Spak (bottom) abundance in the kidneys of WT, Wnk4 D561A/+, Spak −/−, and Wnk4 D561A/+.Spak −/− mice. (PPT) [file pone.0072969.s001.ppt]

## Slide 1
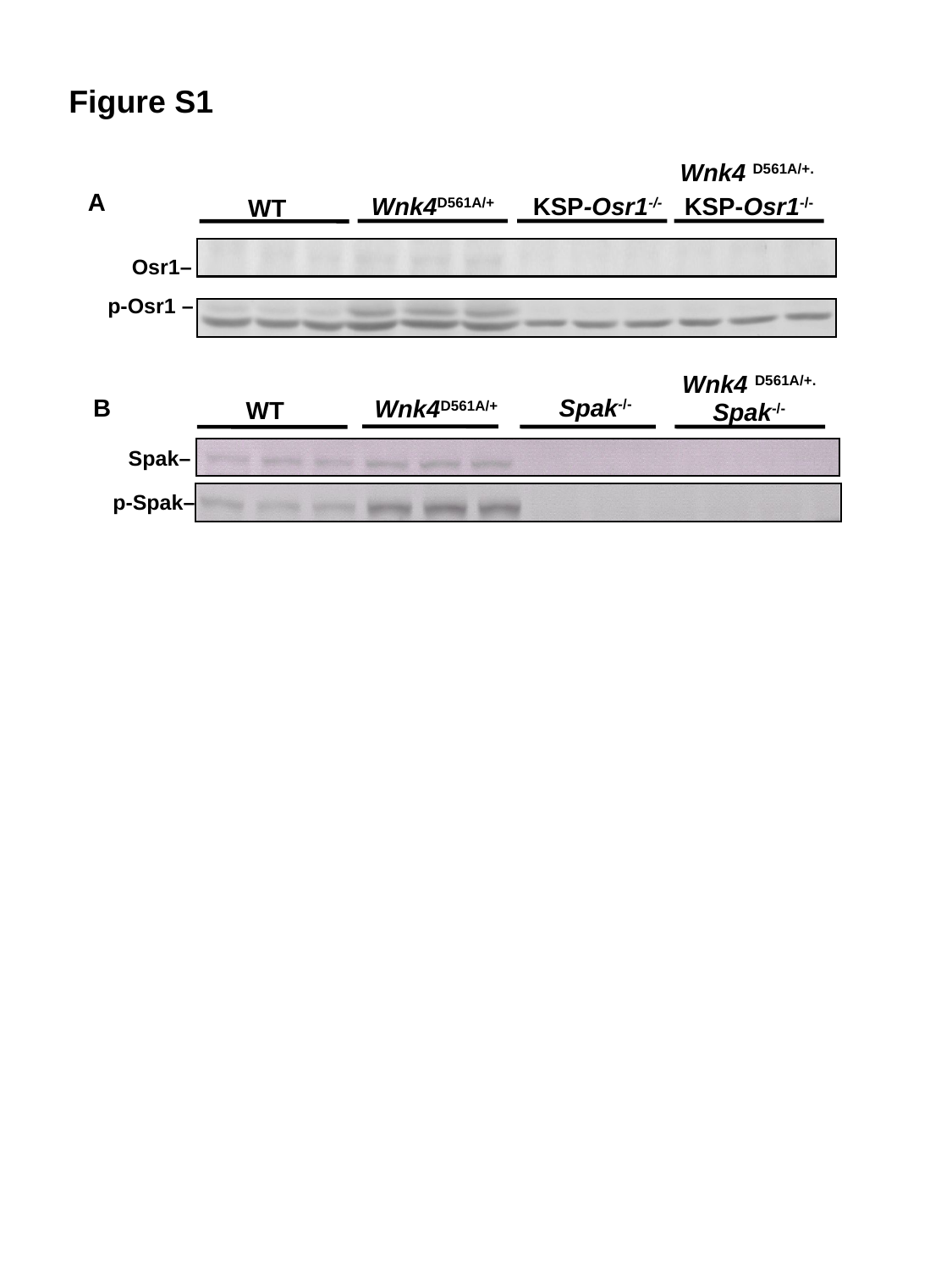

Figure S1
Wnk4 D561A/+.
KSP-Osr1-/-
A
Wnk4D561A/+
 KSP-Osr1-/-
WT
Osr1–
p-Osr1 –
Wnk4 D561A/+.
Spak-/-
B
 Spak-/-
Wnk4D561A/+
WT
Spak–
p-Spak–

## Slide 2
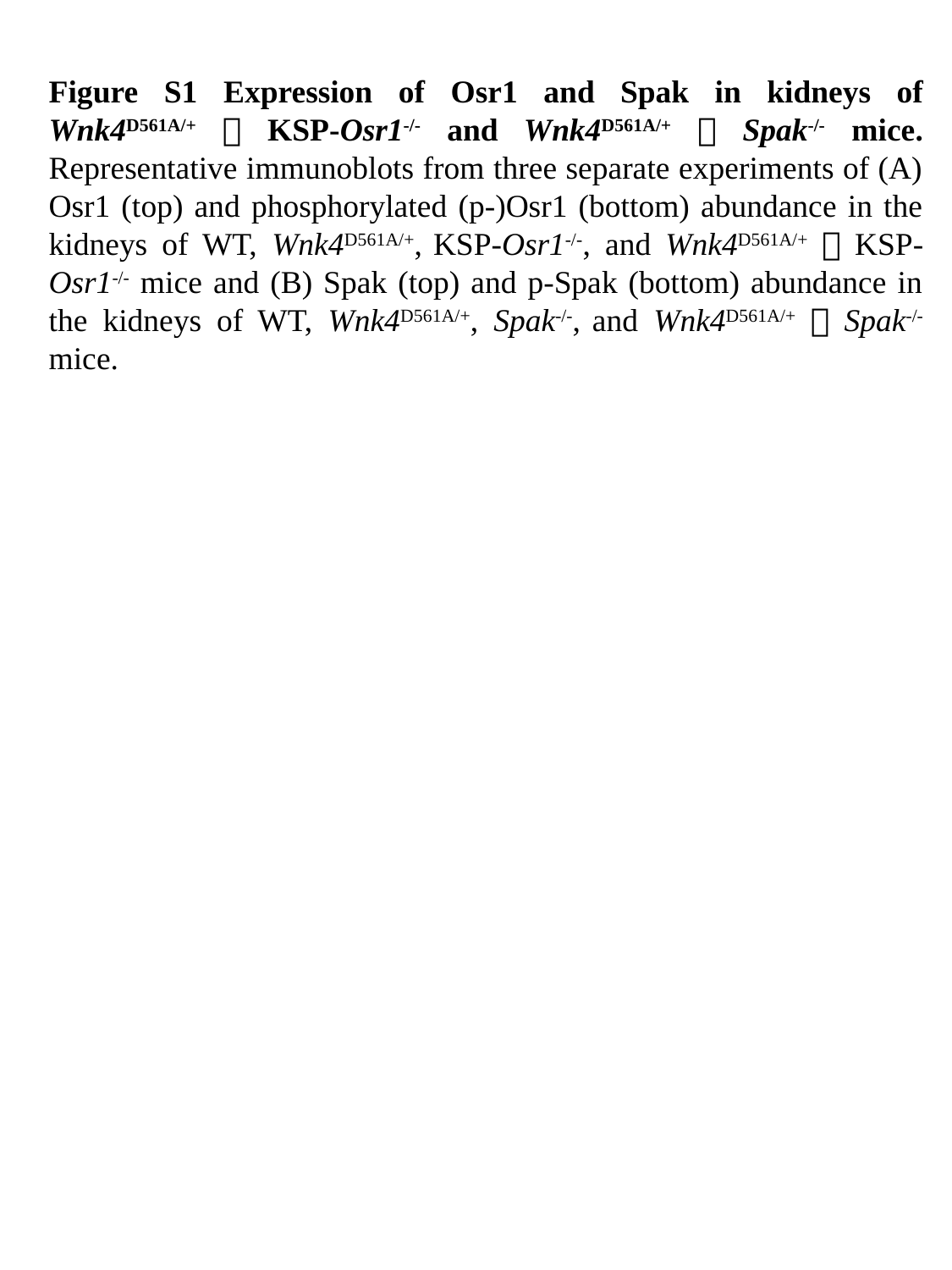

Figure S1 Expression of Osr1 and Spak in kidneys of Wnk4D561A/+．KSP-Osr1-/- and Wnk4D561A/+．Spak-/- mice. Representative immunoblots from three separate experiments of (A) Osr1 (top) and phosphorylated (p-)Osr1 (bottom) abundance in the kidneys of WT, Wnk4D561A/+, KSP-Osr1-/-, and Wnk4D561A/+．KSP-Osr1-/- mice and (B) Spak (top) and p-Spak (bottom) abundance in the kidneys of WT, Wnk4D561A/+, Spak-/-, and Wnk4D561A/+．Spak-/- mice.
