# Supplementary material for: SPAK Deficiency Corrects Pseudohypoaldosteronism II Caused by WNK4 Mutation
Source: PLoS One. 2013 Sep 11;8(9):e72969. doi: 10.1371/journal.pone.0072969 (PMC3770638; doi:10.1371/journal.pone.0072969)
Supplement: Figure S2 — Immunofluorescence images of Nkcc2 and p-Nkcc2(T96) in kidneys of WT and Wnk4 D561A/+mice. In WT mice, Nkcc2 (red) was dominantly expressed in the medullar (M) region and p-Nkcc2 (green) was mostly expressed in the cortical (C) region. In Wnk4 D561A/+ mice, the abundance of Nkcc2 (red) in the medullar region was reduced but p-Nkcc2(green) in the cortical region was enhanced. The scale bars indicate 100 µm. (PPT) [file pone.0072969.s002.ppt]

## Slide 1
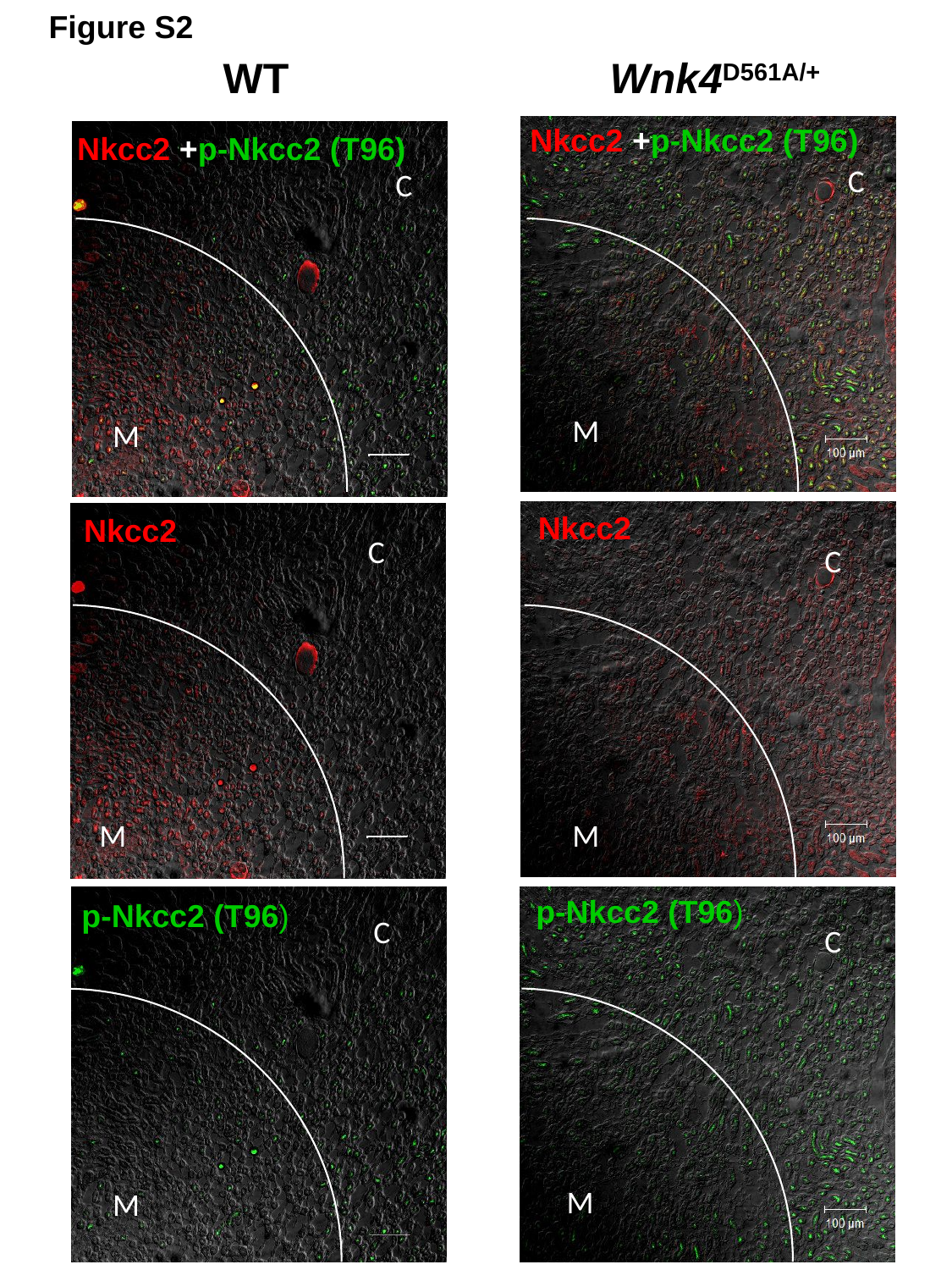

Figure S2
WT
Wnk4D561A/+
Nkcc2 +p-Nkcc2 (T96)
Nkcc2 +p-Nkcc2 (T96)
C
C
M
M
Nkcc2
Nkcc2
C
C
M
M
p-Nkcc2 (T96)
p-Nkcc2 (T96)
C
C
M
M

## Slide 2
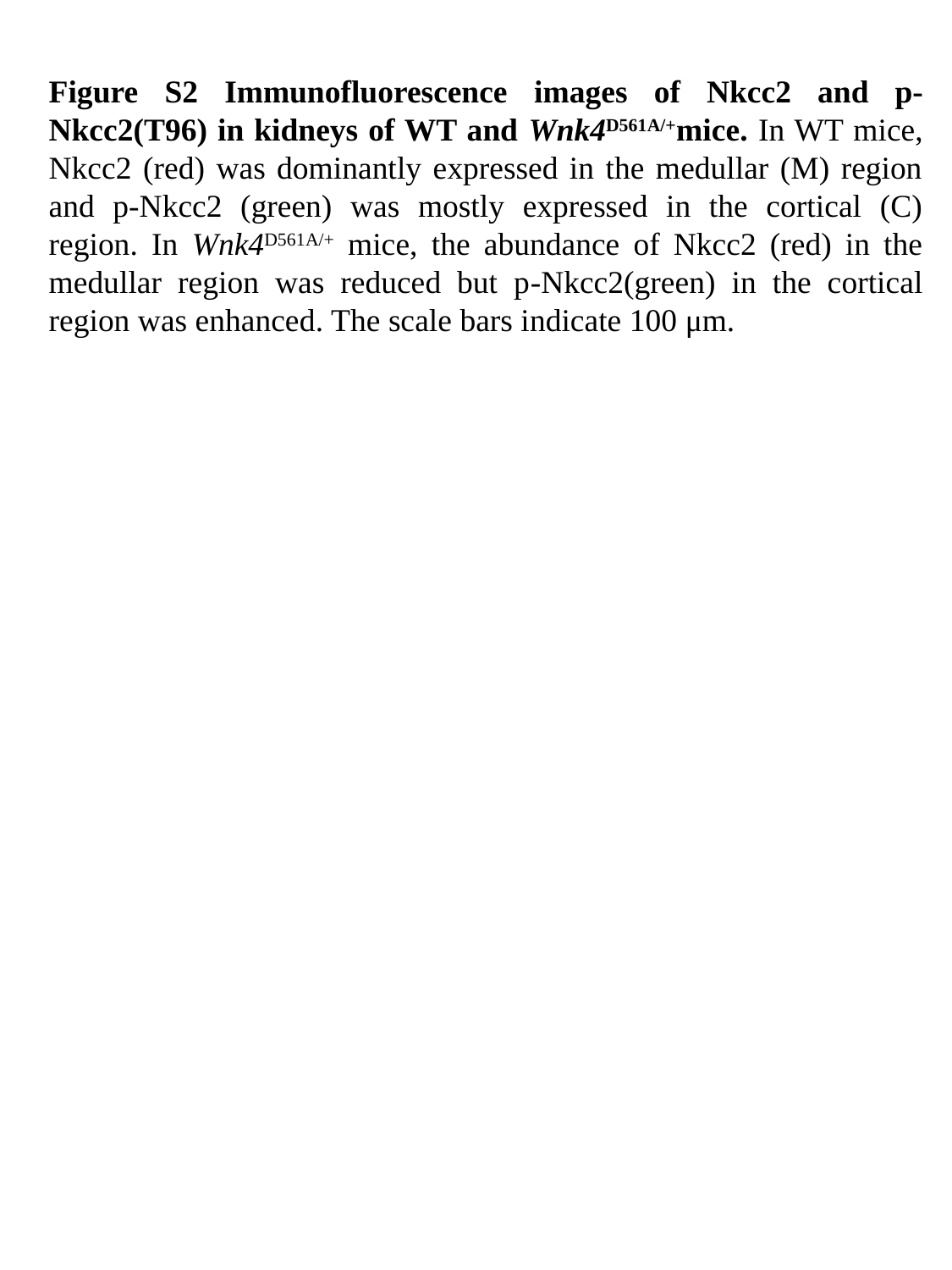

Figure S2 Immunofluorescence images of Nkcc2 and p-Nkcc2(T96) in kidneys of WT and Wnk4D561A/+mice. In WT mice, Nkcc2 (red) was dominantly expressed in the medullar (M) region and p-Nkcc2 (green) was mostly expressed in the cortical (C) region. In Wnk4D561A/+ mice, the abundance of Nkcc2 (red) in the medullar region was reduced but p-Nkcc2(green) in the cortical region was enhanced. The scale bars indicate 100 μm.
