# Supplementary material for: SPAK Deficiency Corrects Pseudohypoaldosteronism II Caused by WNK4 Mutation
Source: PLoS One. 2013 Sep 11;8(9):e72969. doi: 10.1371/journal.pone.0072969 (PMC3770638; doi:10.1371/journal.pone.0072969)
Supplement: Figure S4 — Low-power immunofluorescence of renal Ncc and Nkcc2. (Spak experiment series) Representative (A) total Ncc, (B) p-Ncc (T53), (C) p-Ncc (T58), (D) p-Ncc (S71), (E) total Nkcc2, and (F) p-Nkcc2 (T96) in kidneys of WT, Wnk4 D561A/+, Spak −/−, and Wnk4 D561A/+.Spak −/− mice. The scale bars indicate 100 µm. (PPT) [file pone.0072969.s004.ppt]

## Slide 1
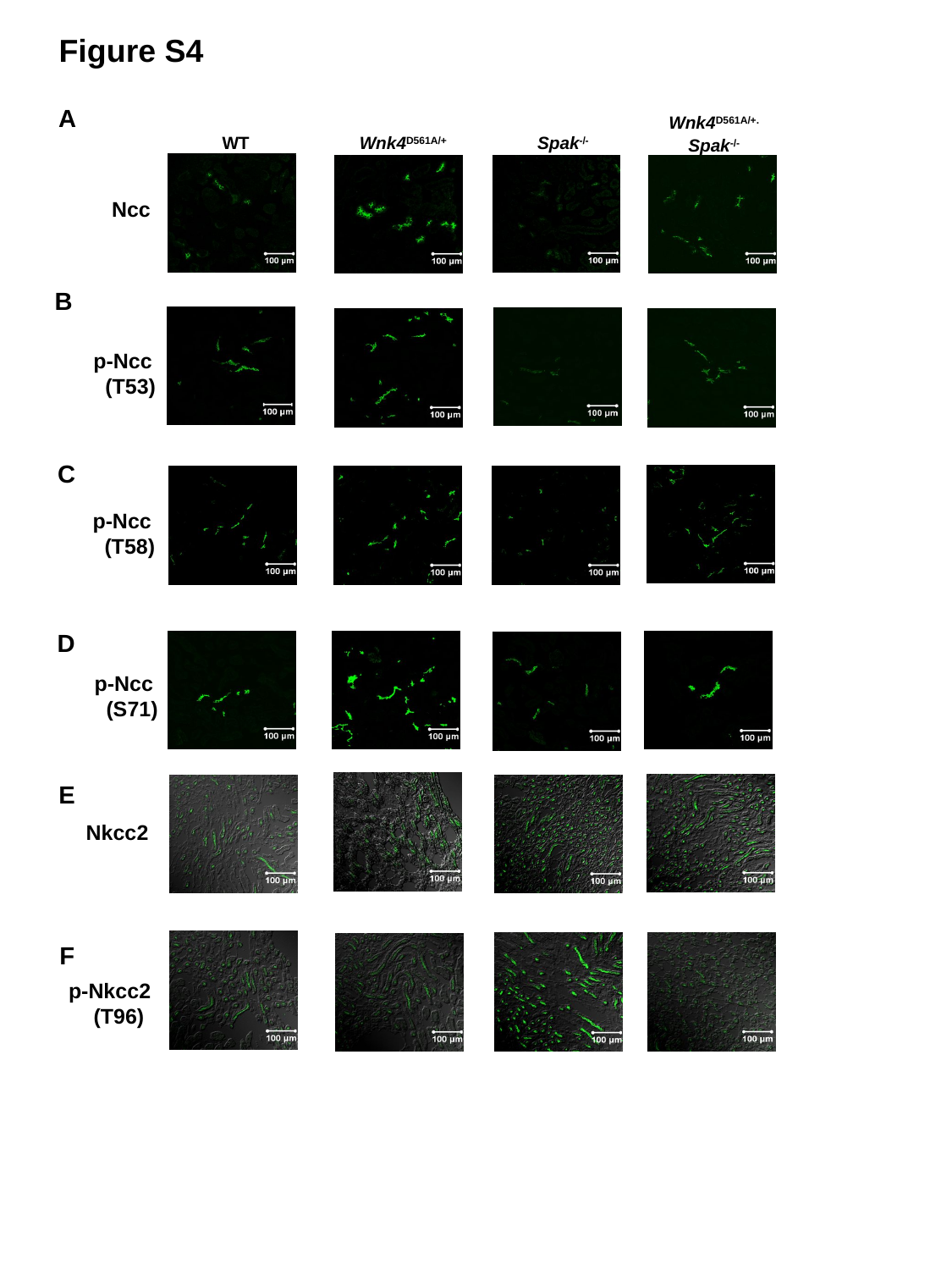

Figure S4
A
Ncc
B
p-Ncc
 (T53)
C
p-Ncc
 (T58)
D
p-Ncc
 (S71)
E
Nkcc2
F
p-Nkcc2
 (T96)
Wnk4D561A/+.
Spak-/-
WT
Wnk4D561A/+
 Spak-/-

## Slide 2
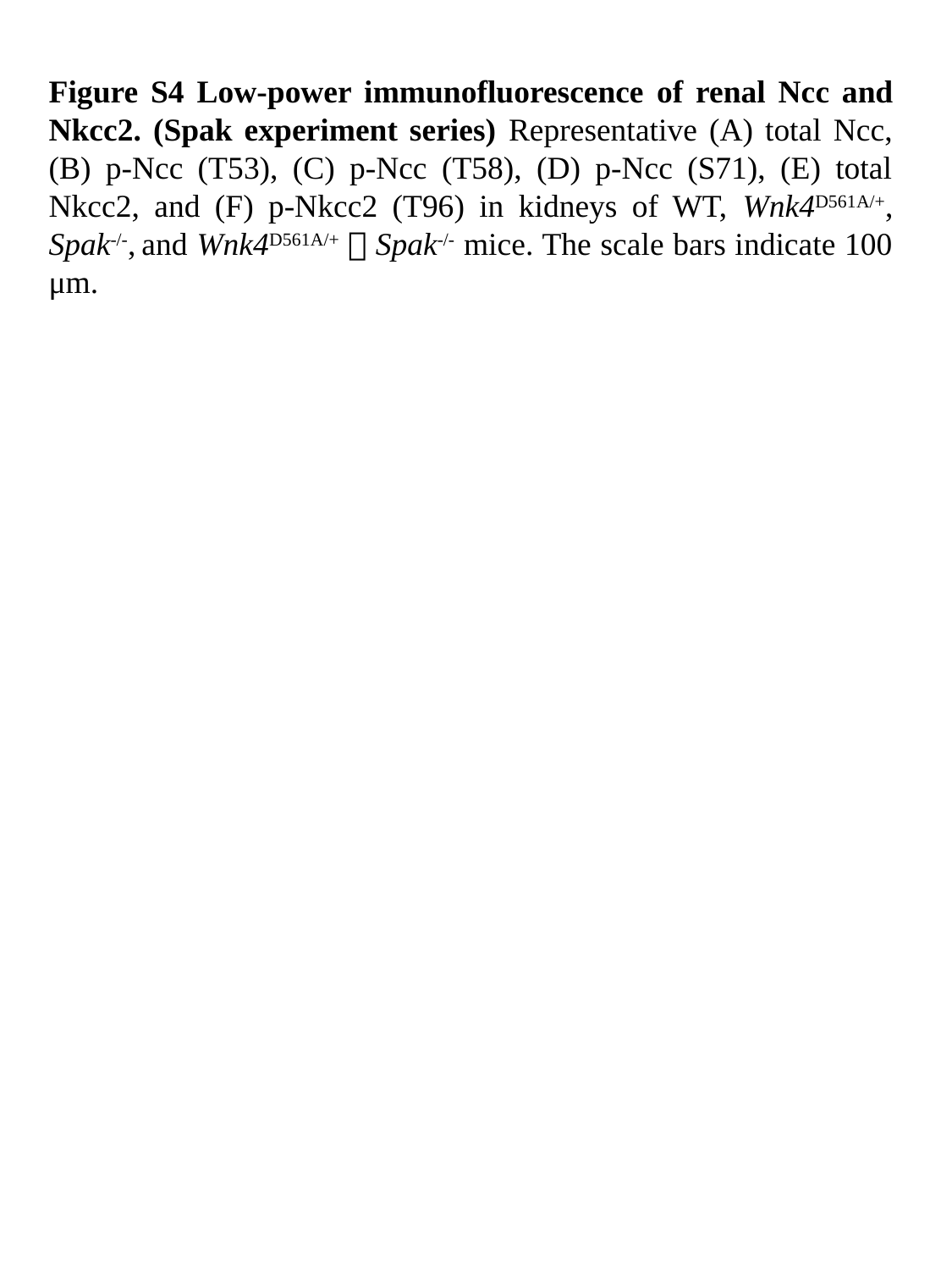

Figure S4 Low-power immunofluorescence of renal Ncc and Nkcc2. (Spak experiment series) Representative (A) total Ncc, (B) p-Ncc (T53), (C) p-Ncc (T58), (D) p-Ncc (S71), (E) total Nkcc2, and (F) p-Nkcc2 (T96) in kidneys of WT, Wnk4D561A/+, Spak-/-, and Wnk4D561A/+．Spak-/- mice. The scale bars indicate 100 μm.
